# Possible Regulation of Larval Juvenile Hormone Titers in *Bombyx mori* by *BmFAMeT6*

**DOI:** 10.3390/insects14070644

**Published:** 2023-07-17

**Authors:** Yang Yu, Tian Li, Meiwei Guo, Rong Xiong, Dongshen Yan, Ping Chen

**Affiliations:** 1 College of Sericulture, Textile and Biomass Sciences, Southwest University, Chongqing 400715, China; yy18223722377@163.com (Y.Y.); litianforever@163.com (T.L.);; 2 Drug Discovery Research Center, Southwest Medical University, Luzhou 646099, China; 3 State Key Laboratory of Silkworm Genome Biology, Southwest University, Chongqing 400715, China

**Keywords:** *Bombyx mori*, *BmFAMeT6*, overexpression, CRISPR/Cas 9, juvenile hormone

## Abstract

**Simple Summary:**

Juvenile hormone (JH) is very important to the growth and development of insects, but the role of farnesoic acid O-methyltransferase (FAMeT) on JH titer has not been determined in insects. In this study, *BmFAMeT6* overexpression and *BmFAMeT6* knockout strains were established by using the Gal4/UAS binary hybrid system and CRISPR/CAS 9 system, respectively. The role of FAMeT in the regulation of insect JH titers and the relationship between farnesoic acid and the synthesis of JH (JH I and JH II) were, first, revealed directly in insects. This provides a new perspective for further understanding the synthesis and regulation of JH in silkworms and other insects.

**Abstract:**

Juvenile hormone (JH) plays a vital role in the growth, development, and reproduction of insects and other arthropods. Previous experiments have suggested that *BmFAMeT6* could affect the duration of the silk moth’s larval stage. In this study, we established the *BmFAMeT6* overexpression strain and *BmFAMeT6* knockout strain using the GAL4/UAS binary hybrid system and CRISPR/Cas 9 system, respectively, and found that the larval stage of the overexpression strain was shorter, while the knockout strain was longer. Our results exhibited that both the JH titers and *BmKr-h1* levels in the larvae of the third instar were reduced significantly by *BmFAMeT6* overexpression, but were increased obviously by *BmFAMeT6* knockout. In addition, injection of farnesoic acid induced changes in the JH I and JH II levels in the hemolymphs of larvae. This study is the first to directly reveal the role of *BmFAMeT6* in the regulation of insect JH titers and the relationship between farnesoic acid and JH (JH I and JH II). This provides a new perspective on regulating the growth and development of insects such as *Bombyx mori*.

## 1. Introduction

### 1.1. Synthesis Pathway of Juvenile Hormone

**Figure 1:**
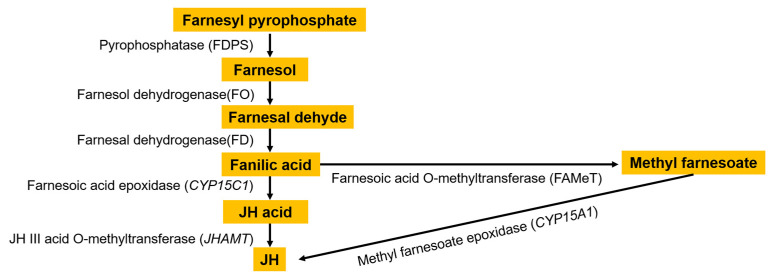
Juvenile hormone synthesis pathway.

The silkworm is an economically important holometabolous insect, and its growth and development are closely related to juvenile hormone (JH). In the adult stage of insects, JH is an essential molecule in the reproductive process, including vitellogenesis, egg maturation, and reproductive behavior, which has been extensively studied at both physiological and molecular levels [1,2]. There are eight types of natural JHs characterized in insects. Only JH Ⅲ is ubiquitous in all insects. Four JH variants, JH0, iso JH0 (also called 4-methyl JH Ⅰ), JH Ⅰ, and JH Ⅱ, have been found exclusively in Lepidoptera [3,4]. The synthesis of JH is divided into the mevalonate (MVA) pathway and the isoprenoid branch [5,6]. Cheng et al. [7] found that the genes related to the upstream seven steps of the MVA pathway of JH biosynthesis, which is responsible for producing isopentenyl pyrophosphate (IPP) as farnesyl diphosphate (FPP) precursor, all existed as a single copy and displayed a rigorous orthologous relationship in a variety of insects. The conversion of farnesoic acid (FA) into JH takes place in two ways [8,9]: (1) cytochrome oxidase catalyzes FA to produce juvenile hormone acid (JHa), which is then catalyzed by JH acid methyltransferase (JHAMT) to produce biologically active JH; (2) the conversion of FA to methyl farnesoate (MF) is catalyzed by farnesoic acid O-methyltransferase (FAMeT), and then cytochrome P450 (CYP15) catalyzes methyl farnesoate (MF) to produce JH (Figure 1).

### 1.2. Role of FAMeT in Juvenile Hormone Synthesis

Deciphering JH signaling in insects has attracted attention worldwide, especially regarding the terminal steps of JH synthesis. There are studies about JHAMT’s role in the JH titer of various species, including the red flour beetle *Tribolium castaneum*, *Bombyx mori*, *Aedes aegypti*, and *Pardosa pseudoannulata* [10,11,12,13]. However, most studies have focused on crustaceans and indicated that FAMeT is closely related to MF synthesis, a process in which methoprene-tolerant (Met) and Kruppel homolog 1 (Kr-h1) are mediated to participate in vitellogenesis, ultimately affecting reproduction [14,15]. The gene encoding FAMeT was first identified in crustaceans [16]. In insects, FAMeT was first reported in *Drosophila melanogaster*, but it did not have significant catalytic activity in transforming FA to MF [17]. However, Vannini et al. suggested that FAMeT might have a functional role in JH biosynthesis after obtaining the gene expression profile in the pre-imaginal life of *Medfly* putatives [18]. And FAMeT has two isoforms in the stingless bee, *Melipona scutellaris*, of which only the expression of isoform 2 seems to be modulated by JH-III [19]. These contradictory results have not yet been resolved, so it is worthwhile to carry out this research in such a powerful model organism as the silkworm. Furthermore, the expression of FAMeT was increased when the *NLKR-h1* gene was knocked out in *Nilaparvata lugens* [20]. Meng et al. [21] identified seven genes (*BmFAMeT1* to *BmFAMeT7*) which encode FAMeT homologues in the silkworm. Decreasing the expression level of *BmFAMeT6* could prolong the larval stage [22]. Based on the above studies, we hypothesized that FAMeT may affect the span of the silkworm larval stage by regulating JH. To validate this hypothesis, we constructed individuals with *BmFAMeT6* overexpression and knockout to directly explore the relationship between FAMeT and JH in the silkworm.

## 2. Materials and Methods

### 2.1. Experimental Insects and Feeding

In the early stage, we used the Dazao (or P50) strain to produce a binary GAL4/UAS system transgenic silkworm using the method of Ma et al. [23]. Thereby, individuals overexpressing the *BmFAMeT6* gene (both DsRed2-positive and EGFP-positive) and single fluorescent (DsRed2-positive or EGFP-positive) individuals were obtained. We used single fluorescent individuals as the control group (Con) and positive individuals as the overexpression group (Ov). Knockout individuals were constructed using CRISPR/Cas 9 techniques in reference to the methods of Dai et al. [24], and single fluorescent (EGFP-positive) individuals were used as the control group (CON) while positive individuals (both DsRed2-positive and EGFP-positive) were used as the knockout group (Ko). The Dazao strain was maintained in our laboratory resource banks at Southwest University (Chongqing, China). Each group had three replicates, and each replicate had at least 200 silkworms. Eggs were kept at 25 °C, with 85 ± 5% humidity until hatching, while larvae (instars Ⅰ–Ⅴ) were fed with fresh mulberry leaves and kept under natural light conditions (25 ± 2 °C and 75 ± 5% relative humidity). The growth and development of *Bombyx mori* were recorded during the experiment.

### 2.2. Fluorescence Screening of Transgenic Silkworm and Insertion Site Detection of Overexpressed Genes

Silkworms were raised to moths and screened with an Olympus SZX12 fluorescence microscope (Olympus, Tokyo, Japan). The overexpressed individuals (both DsRed2-positive and EGFP-positive) were frozen in liquid nitrogen, and their genomes were extracted after grinding. After genome enzyme digestion, purification, and cyclization of enzyme digestion products, reverse PCR was performed using pBacL and pBacR primers (Table S1). Finally, fragments were recovered and connected with the PMD19-T Simple vector. The overexpression insertion sites were analyzed in the SilkMap website (http://www.silkdb.org/silksoft/silkmap.html, accessed on 4 May 2021). The knockout individuals (both DsRed2-positive and EGFP-positive) genomes were extracted and sequenced to compare and analyze the deletion and/or mutation of the bases near the gRNA site of the genome.

### 2.3. Molecular Detection of Transgenic Bombyx Mori

RNA was extracted from the larval head and reverse transcribed into cDNA. The Primer Premier5 software (Primerprimer, Canada) was used to design *BmFAMeT6* and *BmKr-h1* primers (Table S1) for real time-quantitative PCR (RT-qPCR). The silkworm eukaryotic translation initiation factor 4A (silkworm microprobe number: sw22934) was used as an internal control. The reaction conditions were as follows: 95 °C, 30 s (pre-denaturation); 95 °C, 5 s (denaturation); 62 °C, 60 s (annealing); 60 °C, 30 s (extension); 40 cycles. The data were calculated with the 2^−ΔΔCT^ method.

The protein of the heads was extracted in analysis buffer (Thermo Fisher Scientific, Waltham, MA, USA), and the concentration was determined with a BCA kit (Beyotime Bio. Co., Shanghai, China). Finally, the protein level was analyzed by western blot and ImageJ software (National Institutes of Health, Bethesda, MD, USA). The dilution ratios of the primary (Customized Rabbit antibody, Genscript Bio. Co., Nanjing, China) and secondary (Beyotime Bio. Co., Shanghai, China) antibodies were 1:1000 and 1:2000, respectively.

### 2.4. Detection of Farnesoic Acid (FA), Methyl Farnesoate (MF), Juvenile Hormone I and Ⅱ (JH Ⅰ and JH Ⅱ)

The silkworms’ hemolymphs were collected at 0 h (3I0H, 0 h after the second larval molt, namely, the beginning of the third instar), 12 h (3I12H, 12 h after the second molt), and 24 h (3I24H, 24 h after the second molt) of the third instar. First, the hemolymph was sampled by ventral appendage puncture with a sterile needle. Then, 100 μL of hemolymph was added to 5 mL of extraction solution (ether: methanol = 1:1) with a pipette controller, mixed with 2 mL of n-hexane, and centrifuged at 4 °C and 11,200 rpm for 10 min. The supernatant was obtained, and the procedure was repeated three times. Finally, the supernatant was dried with high-purity nitrogen, and JH Ⅰ (overexpression experiment), FA, and MF were analyzed on the HPLC (Thermo, Waltham, MA, USA). Instrument conditions were as follows: chromatographic column (C18), mobile phase (volume of acetonitrile: volume of water = 35:65 or 70:30), flow rate (1 mL/min), wavelength (200 or 225 nm), and column temperature (22 °C). A commercial ELISA kit (Jingmei, Jiangsu, China) was used to detect JH Ⅰ and JH Ⅱ levels (knockout experiment), and high-performance liquid chromatography (HPLC) was used to detect JH precursors (FA and MF) in the hemolymph of third instar silkworms.

### 2.5. Construction of the Recombinant Expression Vector

The first and second exons on the *BmFAMeT6* gene sequences were selected, and the first three gRNA sequences with high scores on the gRNA design website (https://crispr.dbcls.jp/, accessed on 4 May 2021) were selected. We designed the U6-gRNA cloning primer sequence and added the Bg l Ⅱ restriction site to the primer, cloned and ligated to the t-vector, and simultaneously digested the intermediate vector and Piggybac with Bg l Ⅱ [3 × P3-Red] vector. Finally, the U6-gRNA fragment digested from T upload was ligated to Piggybac [3 × P3-Red] final vector for bacterial broth PCR and sequencing validation. The exfoliated epidermis of the transgenic double fluorescent silkworm was collected before it was transformed into the moth, the genome was extracted and sequenced, and the expression level of the target gene was detected by RT-qPCR. The relevant primers are shown in Table S1 (Supplementary Materials).

### 2.6. Injection of Exogenous Farnesoic Acid

Larvae of the last stage of 2nd instar were immobilized by chilling, were injected with 5 μL FA (2 μg/μL) solution (FA-injection) from the third stomata quickly to avoid bleeding as much as possible, and then 5 μL PBS solution was injected as a control under the same conditions (PBS-injection). In order to increase the FA concentration in the hemolymph after injection, we used the method of superposition injection, that is, after the first injection, the superposition injection was performed at 12 h intervals, and the amount of injection was the same as that of the first injection. Then, the hemolymph was collected for JH Ⅰ and JH Ⅱ titer detection by the ELISA kit (Jingmei, Jiangsu, China).

### 2.7. Data Processing

Experimental data were statistically analyzed using Student’s *t*-test. Statistical tests and drawing were conducted with Prism 8.0 (GraphPad Prism Inc., San Diego, CA, USA) software. A *p*-value < 0.05 indicated a significant difference (*), and a *p*-value < 0.01 indicated a highly significant difference (**). *p*-values < 0.001 and <0.0001 are marked by “***” and “****”, respectively.

## 3. Results

### 3.1. Insertion Sites of Overexpressed Genes

**Figure 2:**
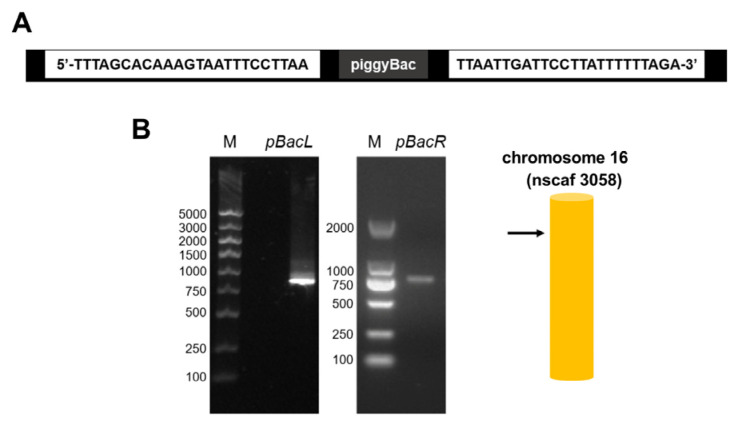
Schematic diagram of the insertion site of the transgenic silkworm. (**A**) 20 bp sequence of the foreign gene at the two ends of the insertion site in the silkworm genome. (**B**) Bands amplified at the pBacL and pBacR arms were obtained using corresponding primer pairs; M: marker. Color patterns were obtained from further sequence alignment and chromosome mapping (SilkMap) analysis based on the sequencing results.

The target gene *BmFAMeT6*, constructed on a piggyBac vector, was successfully integrated into the genome of *Bombyx mori* (Figure 2) using a pBacL arm and pBacR arm (>500 bp), and the insertion site was located on chromosome 16 nscaf 3058 (Figure 2B).

### 3.2. Identification of the Overexpression Silkworm Strain

**Figure 3:**
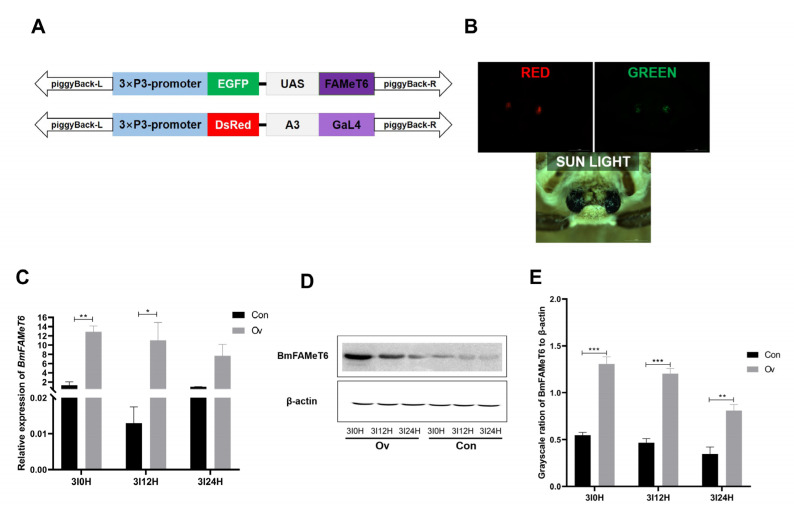
Transgenic individual verification. (**A**) Schematic diagram of the construction of the overexpression plasmid. (**B**) Fluorescent images of the transgenic silkworm in moth stages. (**C**) Expression of *BmFAMeT6* in silkworm heads at instar 3. (**D**) Western blot analysis of BmFAMeT6 in the head. (**E**) Protein band gray analysis of *BmFAMeT6*. Con: control type; Ov: overexpression type. 3I0H: 0 h of the third instar; 3I12H: 12 h of the third instar; 3I24H: 24 h of the third instar. Three replicates were analyzed, and each replicate included at least five samples. Mean ± SEM, N = 20. A *p*-value < 0.05 indicated a significant difference (*), and a *p*-value < 0.01 indicated a highly significant difference (**). *p*-values < 0.001 is marked by “***”.

*BmFAMeT6* was overexpressed by the GAL4/UAS system (Figure 3A). With the help of fluorescence microscopy, both red and green fluorescent proteins were observed in the eyes of overexpressed individuals (Figure 3B). *BmFAMeT6* expression levels at 0 h (3I0H, 0 h after the second larval molt, namely, the beginning of the third instar), 12 h (3I12H, 12 h after the second molt), and 24 h (3I24H, 24 h after the second molt) of the third instar were analyzed by qPCR. In contrast with the Con group, the expression of *BmFAMeT6* in the Ov group was significantly elevated at 3I0H and 3I12H (*p* < 0.05, Figure 3C). The protein level of *BmFAMeT6* was also obviously higher than in the Con group at 3I0H, 3I12H, and 3I24H (*p* < 0.05, Figure 3D,E). Interestingly, the protein and mRNA levels of *BmFAMeT6* decreased gradually with the larval growth from 0H to 24H in the third instar in both the Ov and Con groups (Figure 3C,E).

### 3.3. Construction and Verification of the Knockout Silkworm Strain

**Figure 4:**
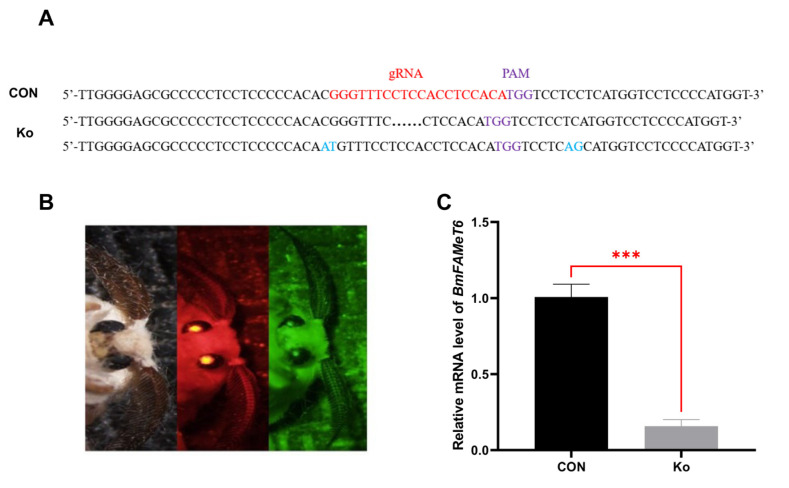
Construction and verification of the knockout silkworm strain. (**A**) Target gene knockout detection. (**B**) Double fluorescence screening of transgenic individuals (from left to right: sunlight, red fluorescence, green fluorescence). (**C**) Expression of *BmFAMeT6* in transgene trains, black points are missing sequences and blue represents the mutated base. CON: control type (green single fluorescent individuals). Ko: knockout type (double fluorescent individuals). Three biological replicates were analyzed, and each replicate included at least three samples. Mean ± SEM, N = 9. *p*-values < 0.001 is marked by “***”.

Individuals with knockout efficiency were found to have base deletions or mutations in the vicinity of genomic gRNA sites (Figure 4A). Target gene expression was detected by RT-qPCR, the results showed that the target gene expression was significantly down-regulated in the individuals with knockout efficiency compared with CON (Figure 4C, *p* < 0.001), indicating that we successfully knocked out the *BmFAMeT6*.

### 3.4. Observation of Phenotype in Transgenic Individuals

**Figure 5:**
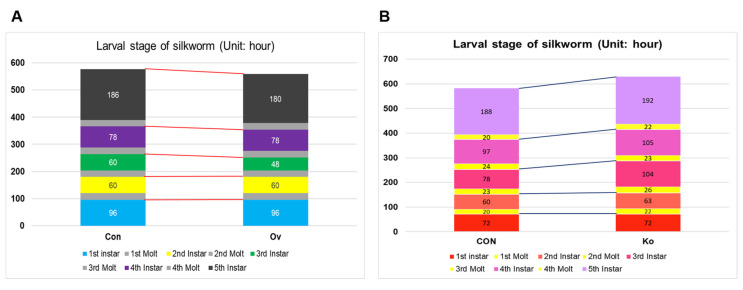
Effects of transgenic individuals on growth in larvae. (**A**) various stages of larval development in OV strain. (**B**) Various stages of larval development in Ko strain. Three replicates were analyzed, and each replicate included at least 25 samples. Mean ± SEM, N = 75. Con: control type; Ov: overexpression type. CON: control type; Ko: knockout type.

The growth process of the larvae was observed, and changes were found in the transgenic silkworm. Specifically, the duration of the third instar was shortened by nearly one day (Figure 5A). Compared with the CON group, *BmFAMeT6* knockout prolonged the larval span of silkworms (byapproximately two days), and the difference was mainly concentrated in the third instar (Figure 5B). At the same time, we measured the body size and weight of the 3I0H, and no significant differences were found.

### 3.5. Analysis of FA and JH Related Indexes in Transgenic Individuals

**Figure 6:**
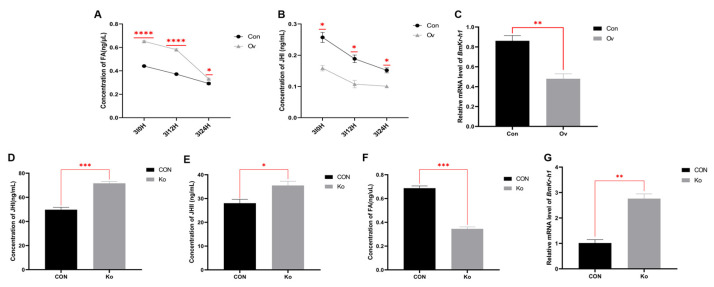
Effects of transgenic individuals on FA- and JH-related indexes in larvae. (**A**) JH Ⅰ concentrations in the third instar between 0 and 24 h. Regression equation: y = 0.0119x + 0.0121, R^2^ = 0.9711; (**B**) FA concentrations. Regression equation: y = 0.3275x − 0.1938, R2 = 0.9971. (**C**) Relative expression levels of BmKr-h1 genes in third instar silkworms. (**D**) JH Ⅰ concentrations at 0 h of the third instar. Regression equation: y = 27.564x − 0.1938, R^2^ = 0.9992; (**E**) JH Ⅱ concentrations at 0 h of the third instar, y = 19.972x − 1.12068, R^2^ = 0.9931. (**F**) FA concentrations at 0 h of the third instar. Regression equation: y = 0.3275x− 0.1938, R2 = 0.9971. (**G**) relative expression levels of genes related to JH synthesis in third instar silkworms. Three replicates were analyzed, and each replicate included at least eight samples. Mean ± SEM, N = 24. Con: control type; Ov: overexpression type. CON: control type; Ko: knockout type. A *p*-value < 0.05 indicated a significant difference (*), and a *p*-value < 0.01 indicated a highly significant difference (**). *p*-values < 0.001 and <0.0001 are marked by “***” and “****”, respectively.

Compared with the Con group, the overexpression of *BmFAMeT6* significantly decreased the JH Ⅰ titer of third instar larvae at three time points (0H, 12H, 24H), but the FA concentration showed an opposite trend (*p* < 0.05, Figure 6A,B). MF was not detected in all silkworms. In addition, the changes in the JH Ⅰ and FA contents in the two groups decreased with the growth of larvae at 0–24 h in the third instar (Figure 6A,B). The expression of *BmKr-h1* in the Ov group was significantly lower than that in Con group (*p* < 0.01, Figure 6C).

Because the JH Ⅰ content in the previously overexpressed individuals continued to decline after 3I0H, we detected JH titers in the knockout individuals only for 3I0H. Compared with the CON group, the titers of JH Ⅰ and JH Ⅱ were significantly increased and *BmKr-h1* expression was significantly up-regulated (*p* < 0.05, Figure 6D,E,G). However, the concentration of FA in the Ko group was significantly lower than that in the CON group (*p* < 0.01, Figure 6F).

### 3.6. Effect of Exogenous Farnesoic Acid on JH Titer

**Figure 7:**
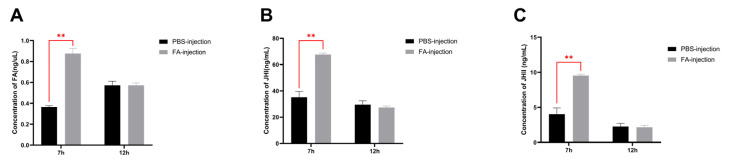
Effects of exogenous farnesoic acid on the JH titer in larvae. (**A**) The FA concentration was 7 h and 12 h after injection, separately. (**B**) The JH Ⅰ concentration was 7 h and 12 h after injection, separately. (**C**) The JH Ⅱ concentration was 7 h and 12 h after injection, separately. Three replicates were analyzed, and each replicate included at least eight samples. Mean ± SEM, N = 48. PBS-injection: 5 uL PBS was injected into wild-type individual. FA-injection: 5 μL FA was injected into wild-type individual. A *p*-value < 0.01 indicated a highly significant difference (**).

Because, in our previous unpublished experimental data, the level of *BmKr-h1* expression in BmN cells was significantly elevated after adding FA to the culture medium, we tested individuals using FA injection. The results showed that, compared with the PBS injection group, the levels of JH Ⅰ, JH Ⅱ, and FA were significantly increased 7 h after the injection of exogenous FA (*p* < 0.01, Figure 7A–C).

## 4. Discussion

To observe the function of the *BmFAMeT6* gene in silkworms, we used an overexpression system to establish *BmFAMeT6* overexpression strains. Stable germline transformation using transposon piggyBac as a transgenic vector has been established for a long time [25]. The GAL4/upstream activation sequence (UAS) binary system in yeast is often used to analyze gene functions by activating or suppressing the expression of target genes in model organisms [26]. The advantage of this system is that the various functions of the target gene in the organism can be observed comprehensively [27]. First generation embryos were screened by observing the fluorescence of DsRed and EGFP, then two single fluorescent individuals were hybridized to obtain double fluorescent positive individuals. The target gene was on the genomic chromosome 16 nscaf 3058, and its mRNA and protein levels were significantly increased in transgenic individuals, indicating that the *BmFAMeT6* gene had been successfully overexpressed. CRISPR-Cas9 has been widely used in biological research since 2013, and its application in *Lepidoptera* insects is also very mature [28]. In order to confirm the effect of *BmFAMeT6* on JH in the overexpression system, we subsequently used CRISPR-Cas9 system to construct knockout individuals, which were found by fluorescence screening to express dual-fluorescent protein, and the deletion and mutation were found by a genome sequencing comparison. Finally, the quantitative analysis also showed that the target gene was down-regulated. These suggested that we successfully constructed the knockout system of the *BmFAMeT6* gene in *Bombyx mori*.

Regarding silkworm growth and development, the spans of the larval stage (instars 3 and 5) of the Ov group were shorter than those of the Con group. These results suggest that *BmFAMeT6* overexpression leads to a shortened larval duration in the silkworm. This is consistent with the results found by Zhang et al. [22]. Specifically, the reduced expression of *BmFAMeT6* through interference transformed a trimolter into a tetramolter, prolonging the larval duration [22]. There was no significant difference in body size and body weight at 3I0H, but the duration of third-instar larva in knockout individuals was prolonged by nearly one day. These results suggested that *BmFAMeT6* has a function in the larval growth of silkworms, and it was confirmed that *BmFAMeT6* mainly affected larval duration by JH titer. The transcription factor Kr-h1, as a key JH signal transducer, reflects the JH signal pathway [29]. Therefore, we analyzed the impact of *BmFAMeT6* on these two indexes (JH and *Kr-h1*) in our follow-up studies.

Through phylogenetic analysis, Meng et al. [21] found that *BmFAMeT6* could be grouped with *Ceratitis capitata* and *Aedes aegypti*. The expression level of FAMeT was consistent with the change in the JH titer in *Ceratitis capitate* [19]. Therefore, we speculated that *BmFAMeT6* in silkworms might also be involved in the titer of JH. Further, *Kr-h1*, as a JH-responsive gene, varies with changes in JH titer, *Kr-h1* provides a good read-out of JH signaling [30,31,32]. Therefore, we analyzed the expression of *BmKr-h1* while detecting the concentration of JH in transgenic silkworms. Moreover, JH is not necessary for first- or second-instar larvae of *Bombyx mori* [4]. Furthermore, Furuta et al. [33] showed that JH Ⅰ and JH Ⅱ are the main juvenile hormone types of silkworm larvae. The titer of JH Ⅰ is significantly higher than that of JH Ⅱ, and the peak of JH Ⅰ appeared at the early stage of the third and fourth instars. Therefore, we measured the JH level of third-instar silkworms within 24 h. In our results, the titer of JH Ⅰ was significantly lower, and the expression level of *BmKr-h1* was also reduced in the Ov group compared to the Con group. This suggests that *BmFAMeT6* overexpression affected the JH titer of the larvae, which was consistent with the span change in the larval stage in the overexpression *BmFAMeT6* strain. In order to further verify the relationship between *BmFAMeT6* and JH, we also did the corresponding index test in the knockout individuals. The results showed that both JH Ⅰ and JH Ⅱ were significantly raised. The *BmKr-h1* expression was significantly up-regulated with the increase in JH. Our work directly proved, for the first time, that *BmFAMeT* regulates the JH titer in insects.

FA is an important precursor for JH III biosynthesis [34]. However, the relationship between FA and JH Ⅰ or JH Ⅱ was unclear in *Lepidoptera*. In the Ov and Con groups, the content of FA and JH Ⅰ decreases with the growth of the larvae at 0–24 H in the third instar. Furthermore, the significant increase in the titers of JH Ⅰ and JH Ⅱ coincides with the FA concentration in the larvae injected with exogenous FA. This demonstrated that FA participate in JH Ⅰ and JH Ⅱ synthesis. Curiously, the change in direction of FA concentration is completely opposite to that of the JH titers in both the *BmFAMeT6* overexpression and *BmFAMeT6* knockout individuality. This may be related to the effects of *BmFAMeT6* on JH synthesis, which may occur in the step from FA to JH. There was a difference between the HPLC and kit in the concentration of JH, which may be caused by the method itself, but the results were consistent.

In crustaceans, the rate-determining step in sesquiterpenoid hormone biosynthesis is the conversion of FA to MF through FAMeT [35]. In this study, we measured FA and MF and found that the concentration of FA, a precursor of JH, was significantly higher in the Ov group than in the Con group at 3I0H. However, MF was not detected in all of the trial silkworms, indicating that the step of FA to MF conversion may not exist in the silkworm. This supports the conclusion that FA is converted into juvenile hormone acid (JHa) by cytochrome oxidase, and then into JH by JH acid methyltransferase (JHAMT) in the final step of insect JH synthesis [36]. Furthermore, studies in *Drosophila melanogaster* have reported that FAMeT cannot convert FA into MF to participate in the biosynthesis of JH [16,17]. The results of our study coincide with this claim. However, how *FAMeT6* affects FA and how it regulates JH titers needs further study.

## 5. Conclusions

We analyzed the overexpression and knockout strains, and found that *BmFAMeT6* affected the JH titer, and injected FA into larvae. As well, we found that FA participate in JH Ⅰ and JH Ⅱ synthesis in the larval stage of silkworms. Our study provides a new perspective for further understanding the synthesis and regulation of JH in silkworms and other insects.

## Data Availability

All the datasets in this study can be provided relay on reasonable request.

## Supplementary Materials

The following supporting information can be downloaded at: https://www.mdpi.com/article/10.3390/insects14070644/s1, Table S1. The primer sequences used in this study.

## References

[1] Daimon T., Uchibori M., Nakao H., Sezutsu H., Shinoda T. (2015). Knockout silkworms reveal a dispensable role for juvenile hormones in holometabolous life cycle. Proc. Natl. Acad. Sci. USA.

[2] Wyatt G.R., Davey K.G. (1996). Cellular and Molecular Actions of Juvenile Hormone. II. Roles of Juvenile Hormone in Adult Insects. ADV Insect Physiol..

[3] Chieka M., Riddiford L.M. (2006). Insect juvenile hormone action as a potential target of pest management. J. Pest..

[4] Daimon T., Shinoda T. (2013). Function, diversity, and application of insect juvenile hormone epoxidases (CYP15). Biotechnol. Appl. Biochem..

[5] Bellés X., Martín D., Piulachs M.D. (2005). The mevalonate pathway and the synthesis of juvenile hormone in insects. Annu. Rev. Entomol..

[6] Goodman W.G., Cusson M. (2012). The Juvenile Hormones. Insect Endocrinology.

[7] Cheng D., Meng M., Peng J., Qian W., Kang L., Xia Q. (2014). Genome-wide comparison of genes involved in the biosynthesis, metabolism, and signaling of juvenile hormone between silkworm and other insects. Genet. Mol. Biol..

[8] Hammock B.D. (1975). NADPH dependent epoxidation of methyl farnesoate to juvenile hormone in the cockroach *Blaberus giganteus* L. Life Sci..

[9] Dominguez C.V., Maestro J.L. (2018). Expression of juvenile hormone acid O-methyltransferase and juvenile hormone synthesis in *Blattella germanica*. Insect Sci..

[10] Guo P., Zhang Y., Zhang L., Xu H., Zhang H., Wang Z., Jiang Y., Molloy D., Zhao P., Xia Q. (2021). Structural basis for juvenile hormone biosynthesis by the juvenile hormone acid methyltransferase. J. Biol. Chem..

[11] Minakuchi C., Namiki T., Yoshiyama M., Shinoda T. (2008). RNAi-mediated knockdown of juvenile hormone acid O-methyltransferase gene causes precocious metamorphosis in the red flour beetle *Tribolium castaneum*. FEBS J..

[12] Nouzova M., Edwards M.J., Michalkova V., Ramirez C.E., Ruiz M., Areiza M., DeGennaro M., Fernandez-Lima F., Feyereisen R., Jindra M. (2021). Epoxidation of juvenile hormone was a key innovation improving insect reproductive fitness. Proc. Natl. Acad. Sci. USA.

[13] Yang Z.M., Wu Y., Li F.F., Zhou Z.J., Yu N., Liu Z.W. (2021). Genomic Identification and Functional Analysis of JHAMTs in the Pond Wolf Spider, *Pardosa pseudoannulata*. Int. J. Mol. Sci..

[14] Silva Gunawardene Y.I., Chow B.K., He J.G., Chan S.M. (2001). The shrimp FAMeT cDNA is encoded for a putative enzyme involved in the methylfarnesoate (MF) biosynthetic pathway and is temporally expressed in the eyestalk of different sexes. Insect Biochem. Mol. Biol..

[15] Xie X., Zhu D., Li Y., Qiu X., Cui X., Tang J. (2015). Hemolymph Levels of Methyl Farnesoate During Ovarian Development of the Swimming Crab *Portunus trituberculatus*, and Its Relation to Transcript Levels of HMG-CoA Reductase and Farnesoic Acid O-Methyltransferase. Biol. Bull..

[16] Burtenshaw S.M., Su P.P., Zhang J.R., Tobe S.S., Dayton L., Bendena W.G. (2008). A putative farnesoic acid O-methyltransferase (FAMeT) orthologue in *Drosophila melanogaster* (CG10527): Relationship to juvenile hormone biosynthesis?. Peptides.

[17] Zhang H., Tian L., Tobe S., Xiong Y., Wang S., Lin X., Liu Y., Bendena W., Li S., Zhang Y.Q. (2010). Drosophila CG10527 mutants are resistant to juvenile hormone and its analog methoprene. Biochem. Biophys. Res. Commun..

[18] Vannini L., Ciolfi S., Spinsanti G., Panti C., Frati F., Dallai R. (2010). The putative-farnesoic acid O-methyl transferase (FAMeT) gene of Ceratitis capitata: Characterization and pre-imaginal life expression. Arch. Insect Biochem. Physiol..

[19] Vieira C.U., Bonetti A.M., Simões Z.L., Maranhão A.Q., Costa C.S., Costa M.C., Siquieroli A.C., Nunes F.M. (2008). Farnesoic acid O-methyl transferase (FAMeT) isoforms: Conserved traits and gene expression patterns related to caste differentiation in the stingless bee, *Melipona scutellaris*. Arch. Insect Biochem. Physiol..

[20] Minna J., Yun Y., Bing S., Bo W., Xinda L. (2013). Expression of FAMeT and JHE Influenced by Kruppel homolog-1 Gene from Brown Planthopper. Plant Dis. Pests.

[21] Meng M. (2013). Identification and Expression Profiling of Farnesoic Acid O-methyltrans-ferase Gene in Silkworm, *Bombyx mori*. Sci. Seric..

[22] Zhang L., Li X.Z., Li T., Xiong R., Li Y., Yan D.S., Chen P. (2021). Farnesoic acid methyltransferase 6 (BmFAMeT6) interrelates with moltinism of dominant trimolter in silkworm, *Bombyx mori*. Biologia.

[23] Ma L., Xu H., Zhu J., Ma S., Liu Y., Jiang R.J., Xia Q., Li S. (2011). Ras1(CA) overexpression in the posterior silk gland improves silk yield. Cell Res..

[24] Tan D., Hu H., Tong X., Han M., Gai T., Lou J., Yan Z., Xiong G., Lu C., Dai F. (2022). Mutation of a lepidopteran-specific PMP-like protein, BmLSPMP-like, induces a stick body shape in silkworm, *Bombyx mori*. Pest. Manag. Sci..

[25] Giniger E., Varnum S.M., Ptashne M. (1985). Specific DNA binding of GAL4, a positive regulatory protein of yeast. Cell.

[26] Imamura M., Nakai J., Inoue S., Quan G.X., Kanda T., Tamura T. (2003). Targeted gene expression using the GAL4/UAS system in the silkworm *Bombyx mori*. Genetics.

[27] Duffy J.B. (2002). GAL4 system in *Drosophila*: A fly geneticist’s Swiss army knife. Genesis.

[28] Li J.J., Shi Y., Wu J.N., Li H., Smagghe G., Liu T.X. (2021). CRISPR/Cas9 in lepidopteran insects: Progress, application and prospects. J. Insect Physiol..

[29] Zhang T., Song W., Li Z., Qian W., Wei L., Yang Y., Wang W., Zhou X., Meng M., Peng J. (2018). Krüppel homolog 1 represses insect ecdysone biosynthesis by directly inhibiting the transcription of steroidogenic enzymes. Proc. Natl. Acad. Sci. USA.

[30] Cui Y., Sui Y., Xu J., Zhu F., Palli S.R. (2014). Juvenile hormone regulates Aedes aegypti Krüppel homolog 1 through a conserved E box motif. Insect Biochem. Mol. Biol..

[31] Shinoda T., Itoyama K. (2003). Juvenile hormone acid methyltransferase: A key regulatory enzyme for insect metamorphosis. Proc. Natl. Acad. Sci. USA.

[32] Li K., Jia Q.Q., Li S. (2019). Juvenile hormone signaling—A mini review. Insect Sci..

[33] Furuta K., Ichikawa A., Murata M., Kuwano E., Shinoda T., Shiotsuki T. (2013). Determination by LC-MS of juvenile hormone titers in hemolymph of the silkworm, *Bombyx mori*. Biosci. Biotechnol. Biochem..

[34] Minakuchi C., Ishii F., Washidu Y., Ichikawa A., Tanaka T., Miura K., Shinoda T. (2015). Expressional and functional analysis of CYP15A1, a juvenile hormone epoxidase, in the red flour beetle *Tribolium castaneum*. J. Insect Physiol..

[35] Hui J.H., Hayward A., Bendena W.G., Takahashi T., Tobe S.S. (2010). Evolution and functional divergence of enzymes involved in sesquiterpenoid hormone biosynthesis in crustaceans and insects. Peptides.

[36] Zhang L., Xu H., Zhang Y., Zhang H., Wang Z., Guo P., Zhao P. (2022). Structural characterization and functional analysis of juvenile hormone acid methyltransferase JHAMT3 from the silkworm, *Bombyx mori*. Insect Biochem. Mol. Biol..

