# Supplementary material for: Possible Regulation of Larval Juvenile Hormone Titers in Bombyx mori by BmFAMeT6
Source: Insects. 2023 Jul 17;14(7):644. doi: 10.3390/insects14070644 (PMC10380277; doi:10.3390/insects14070644)
Supplement: Supplementary file 1 [file insects-14-00644-s001.zip › insects-2474421-supplementary.pdf]

Table S1. Primer sequences.

Name	Sequences（5′→3′）
pBacL	F: CTTGACCTTGCCACAGAGGACTATTAGAGG<br>R: CAGTGACACTTACCGCATTGACAAGCACGC
pBacR	F: CGATAAAACACATGCGTCAATTTTACGC<br>R: GCTCTCGACAAATAACTTTTTTGCAT
BmFAMeT6	F: TCCTGTCATAATGCCAACCCC<br>R: TCATTTGCTACCGTGGTCCTC
BmFAMeT6 gRNA1	GGGTTTCCTCCACCTCCACA
BmFAMeT6 gRNA2	GGCGGATTCTGTGCAAGTGG
BmFAMeT6 gRNA3	GAAGCGGTCCCTGAATTACC
Kr-h1	F: ATGATAGGTGACGAGGAGCG<br>R: CGGCAGCCATTGGTGTT
SW22934	F: TTCGTACTGGCTCTTCTCGT<br>R: CAAAGTTGATAGCAATTCCCT
